# Insights into Comparative Modeling of V_H_H Domains

**DOI:** 10.3390/ijms22189771

**Published:** 2021-09-09

**Authors:** Akhila Melarkode Vattekatte, Frédéric Cadet, Jean-Christophe Gelly, Alexandre G. de Brevern

**Affiliations:** 1 Biologie Intégrée du Globule Rouge UMR_S1134, Inserm, Laboratoire d’Excellence GR-Ex, Université de la Réunion, F-97715 Saint Denis Messag, France; akhila.melarkode-vattekatte@univ-reunion.fr (A.M.V.); frederic.cadet.run@gmail.com (F.C.); 2 Biologie Intégrée du Globule Rouge UMR_S1134, Inserm, Laboratoire d’Excellence GR-Ex, Université de Paris, F-75739 Paris, France; jean-christophe.gelly@univ-paris-diderot.fr

**Keywords:** nanobodies, protein structure, homology modeling, secondary structures, structural alphabet

## Abstract

In the particular case of the *Camelidae* family, immunoglobulin proteins have evolved into a unique and more simplified architecture with only heavy chains. The variable domains of these chains, named V_H_Hs, have a number of Complementary Determining Regions (CDRs) reduced by half, and can function as single domains making them good candidates for molecular tools. 3D structure prediction of these domains is a beneficial and advantageous step to advance their developability as molecular tools. Nonetheless, the conformations of CDRs loops in these domains remain difficult to predict due to their higher conformational diversity. In addition to CDRs loop diversity, our earlier study has established that Framework Regions (FRs) are also not entirely conformationally conserved which establishes a need for more rigorous analyses of these regions that could assist in template selection. In the current study, V_H_Hs models using different template selection strategies for comparative modeling using Modeller have been extensively assessed. This study analyses the conformational changes in both CDRs and FRs using an original strategy of conformational discretization based on a structural alphabet. Conformational sampling in selected cases is precisely reported. Some interesting outcomes of the structural analyses of models also draw attention towards the distinct difficulty in 3D structure prediction of V_H_H domains.

## 1. Introduction

**Figure 1:**
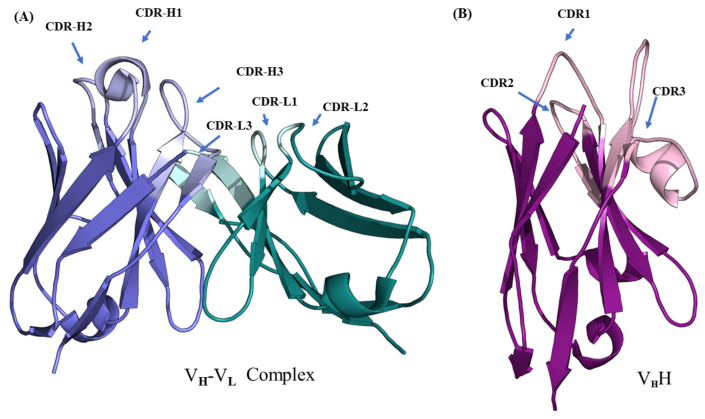
Structural representation of variable regions of immunoglobulins. (**A**) VH–VL complex (taken from PDB ID 4P49 [1]) and (**B**) V_H_H (taken from PDB ID 1JTO [5]). The lighter colored regions represent complementarity determining regions (named CDR-H and CDR-L for the VH-VL complex and CDR for V_H_H) and darker colored regions the framework regions in each domain.

Immunoglobulins or antibodies are crucial proteins of the immune system in jawed vertebrates. Their function is to bind antigens. Amongst the large family of immunoglobulin, immunoglobulin gamma (IgG) has been extensively analyzed, as it is more abundant compared to the rest of the isoforms. IgG is composed of two heavy and two light chains, comprised of approximately 3000 residues organized into multiple regions. Each heavy chain is folded into three conserved domains and one variable domain, and each light chain, into one variable domain and one conserved domain. All the domains have the characteristic immunoglobulin fold. In the N-terminal, domains display larger sequence variability compared to the succeeding domains in each chain; hence, they are called the variable domains (V_H_ and V_L_ of the heavy and light chains). The rest of the sequence is organized into successive conserved domains (C_L_ in light chain and C_H1_, C_H2_, C_H3_, etc., in the heavy chain). The immunoglobulin fold in variable domains is formed by the arrangement of nine antiparallel β-strands (as opposed to 7 β-strands in the conserved domains) connected by loops and arranged into two β-sheets (see Figure 1A [1]). Variable domains contain antigen-binding regions predominantly formed by the loops towards the N-terminus. Belonging to the family of V-set domains in the immunoglobulin superfamily (named IgSF), these variable domains, both V_H_ and V_L_, are composed of two regions: (i) the framework regions (FRs) that correspond mainly to the β-strands, and (ii) the complementarity determining regions (CDRs) composed of the exposed loops, which bind antigenic epitopes. The CDRs exhibit the largest variability in terms of sequence, length, and composition of amino acids, resulting in conformational variability. This large diversity explains the ability of the antibodies to recognize a large number of epitopes. Due to their sensitivity and specificity to bind specific antigens, antibodies have become useful molecules for biotechnological and pharmaceuticals development [2]. Such applications require antibody engineering to improve binding affinity, facilitate humanization process, increase solubility, and alter other biophysical and biochemical properties. Knowledge of the immunoglobulin 3D structure is therefore of great interest, in order to increase their developability. Unfortunately, the availability of large antibody structures is rather limited. Thus, 3D structure prediction of antibody structure is of crucial importance for the development of applications.

The heavy chain only antibodies (HCAbs) from the *Camelidae* family [3,4] are an interesting class of IgGs that completely devoid of light chains. In camelids, they occur in addition to classical IgGs. Due to a specific mutation during the course of evolution, they have lost the entire light chain and C_H_1 domain of the heavy chain. Thus, they bind to antigens using only one variable domain (named V_H_H for the V_H_ domain from HCAb, see Figure 1B [5]). These domains are 120 to 130 residues in length; retain their ability to bind antigens even when expressed independently. This, along with their unique biophysical and biochemical properties, have made them therapeutic, diagnostic, and biotechnological tools [6,7,8]. Due to their increasing applications, these domains need to be engineered to improve their physiochemical properties. Structure prediction of these domains during V_H_H design process can save time and resources. Although the complexity of their 3D structure prediction is slightly reduced in terms of the number of domains to be modeled and the subsequent prediction of their relative orientations, V_H_H has a CDR3 loop that is much more diverse compared to its counterpart in canonical antibodies [9].

The structure prediction of antigen-binding domains for classical antibodies (see Figure 1A) mainly focuses on the two variable domains (V_H_ from the heavy chain and V_L_ from the light chain), each domain comprising four FRs and three CDRs. Although, it is believed/accepted so far that the structure prediction of FRs is straightforward, the CDRs pose a non-trivial task for structure prediction. Another specific difficulty is to predict their relative orientations, which was attempted in recent studies [10,11]. Structure prediction of variable domains, in general, does not specifically require de novo/ab initio methods, as a large number of structures were resolved and deposited in the Protein Data Bank (PDB) [12]. Thus, a typical template-based structure prediction method should be able to generate structural models for these domains. The main difficulty in modeling IgG antibody variable domains lies rather in specific topology of each CDR and the orientations between the two V-set domains to obtain a high accuracy model.

To address this question, a consortium, called the antibody modeling assessment (AMA), exclusively dedicated to IgG structure prediction, was organized twice in the recent past. In these assessment challenges, algorithms, such as RosettaAntibody [13], antibody modeling of Discovery Studio (Accelrys, later BIOVIA from Dassault Systèmes) [14,15], antibody design of the Chemical Computing Group (CCG), and prediction of immunoglobulin structure (PIGS) [16,17] participated in the first evaluation meeting (AMA-I) [18]. The second evaluation (AMA-II) [19] included with the aforementioned four algorithms, are four others groups from Schrödinger, Macromoltek, and a collaborative group by Osaka University and Astellas Pharma. These algorithms differ in their model scorings and refinement protocols, but all of them use template-based modeling, at least to predict the FRs. In both of these assessments, the root mean square deviation (RMSD) was adopted as the gold standard to assess the structural similarity and quality of the models. Most algorithms performed well in the FR prediction with a combined average RMSD range of 0.9 Å ± 0.2 for the domains in the dataset tested. The heavy chain CDR3 remained the most difficult one, with a combined average RMSD of 2.8 Å ± 0.4. The assessments by the two AMAs represent the current state-of-the-art antibody modeling tools [18,19,20]. 

Both AMA assessments did not include any camelid antibody variable domains (V_H_H) in their evaluations. Although some of the algorithms could predict V_H_H structural models, in our literature survey, the generic structure prediction methods are often used. While using comparative modeling, template backbone conformations are critical in determining the protein conformations in the query model, for this reason, a template with best sequence identity to the query was selected to model. In some cases, two or more overlapping templates are used to sample conformational space more exhaustively as seen in few structure prediction studies of V_H_H. In our previous study [21], we observed few examples where the choice of the templates was critical and the variability in sequence identity between FRs and CDRs could be quite complex to handle, e.g., the choice of the structural template with the best sequence identity with query sequence may not always be the best option.

**Figure 2:**
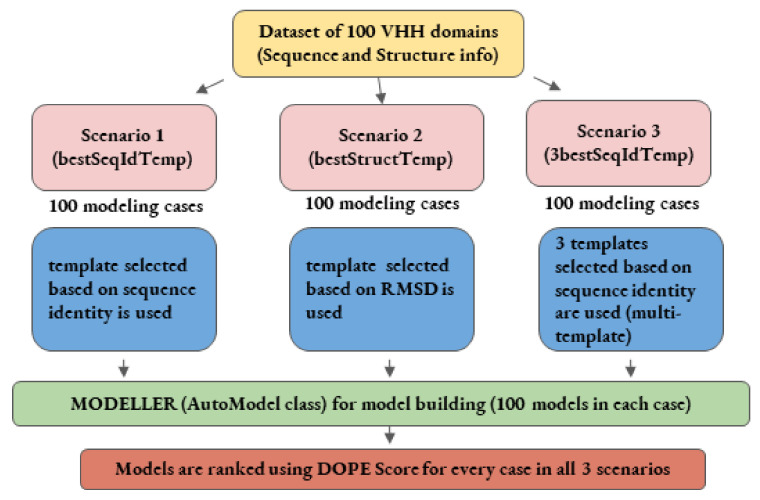
Schematic representation of comparative modeling workflow employed in the first three scenarios.

This study addresses the problem of the impact of template conformations on the V_H_H domain modeling, especially when using single and multi-templates (see Figure 2). It attempts to underline the effect of sequence identity and structural similarity in different regions of these domains, which affect model backbone conformations. Towards this goal, the most extensive dataset of solved V_H_H domains was assembled. Each V_H_H sequence of the dataset was then used blindly to perform comparative modeling using template(s) selected through pairwise sequence identity or structural similarity. Four different scenarios of modeling were assessed to model each query sequence in the dataset; (i) using the best sequence identity template (*bestSeqIdTemp*); (ii) using the best structural template (*bestStructTemp*); (iii) using three best sequence identity templates in a multiple template mode (*3bestSeqIdTemp*) and, finally, as a gold standard, to evaluate the maximal reachable accuracy; (iv) all potential structural templates were tested (*All*). As the AMA competitions, the structural similarity was assessed using RMSD of Cα residues of the best models to the reference native structures. In addition, protein blocks [22,23] were used to measure differences in protein local conformations. As expected, using sequence identity to choose the better template is, most of the time, a reasonable choice. However, for some complex cases, we also highlight that selection of the template based on sequence identity is the worse choice, and identify some V_H_H domains that cannot be properly modeled. Finally, this study presents interesting perspectives that will enable the development of better strategies for V_H_H modeling.

## 2. Results

### 2.1. Selection of V_H_H Sequences

**Figure A1:**
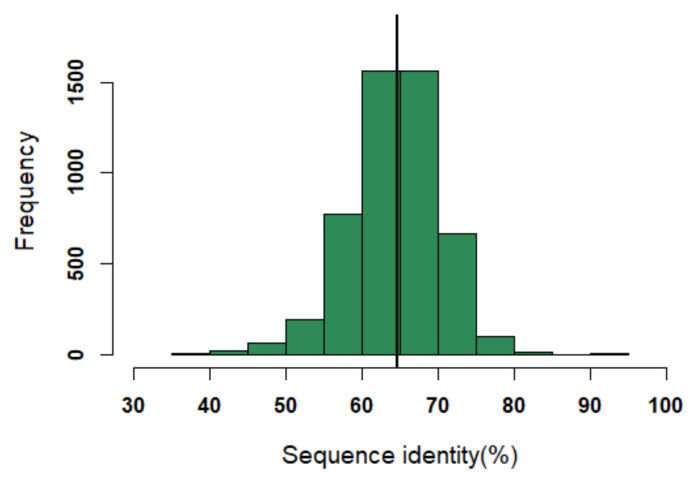
Distribution of sequence identity in the dataset. The black line indicates the median value of the distribution (i.e., sequence identity of 64%).

An initial dataset of 140 PDB entries of V_H_H domains were retrieved from the PDB [12] in a similar way to our previous research [18]. A non-redundant dataset of 125 domains was selected. Out of which, 25 had missing structural data for few residues and, hence, were removed. The final structural dataset consisted of 100 structures. The sequence identity threshold may seem high, but it is a reflection of the highly conserved FRs; closely related FRs can, structurally, be very different [21]. On average, the sequence identity between V_H_H in this structural dataset is only of 64% (see Figure A1).

### 2.2. Global Assessment of Structural Models

#### 2.2.1. V_H_H Models

**Figure 3:**
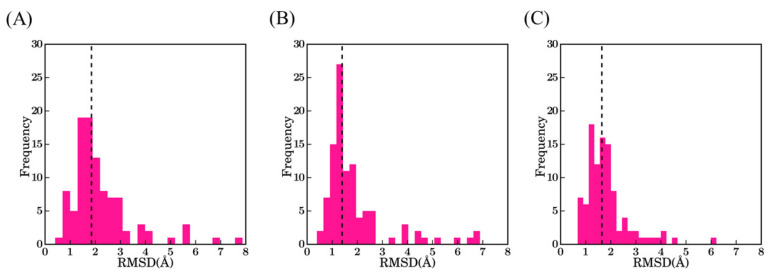
RMSD values between the true V_H_H structure and its best-selected models. (**A**) Scenario 1—*bestSeqIdTemp*; (**B**) scenario 2—*bestStructTemp*; and (**C**) scenario 3—*3bestSeqIdTemp*. The black dotted line indicates the median value of the distributions 1.9 Å, 1.4 Å, and 1.7 Å, respectively.

We generated 100 models for each V_H_H with Modeller [24,25]. Models were ranked accordingly to their predicted quality determined by the DOPE score [26]. Structural similarity of these models to the originally resolved crystal structures was quantified using root mean square deviation (RMSD). Figure 3 represents the structural similarity of best models for the first three scenarios, which are (1) using the best sequence identity template (namely scenario *bestSeqIdTemp*); (2) lowest RMSD template (namely scenario *bestStructTemp*); and (3) multiple template (i.e., three templates that are close in terms of highest sequence identity to the query, namely scenario *3bestSeqIdTemp*). The median RMSD values for the three cases in the ascending order are scenario 2—*bestStructTemp* (1.4 Å) < scenario 3—*3bestSeqIdTemp* (1.6 Å) < scenario 1—*bestSeqIdTemp* (1.9 Å). For the three scenarios, the majority of the best-selected models are at least less than 2.0 Å from the native structure (76% cases for *bestStructTemp*, 74% cases for *3bestSeqIdTemp,* and 56% for *bestSeqIdTemp*). On the contrary, some proposed structural models are of low accuracy. Hence, 9% of *bestStructTemp*, 12% of *3bestSeqtIdTemp,* and 14% of *bestSeqIdTemp* have a RMSD value higher than 3.0 Å. This result may seem surprising in comparison to the simplicity of immunoglobulin fold, but the same was also observed during multiple previous analyses [27,28]

Only 11 V_H_Hs share the same template for scenario 1 and 2, i.e., the best sequence identity is also the closest in terms of RMSD. Thus, to summarize, using only the sequence identity, the addition of two other templates provides an average gain of 0.2 Å (*bestSeqIdTemp* and *3bestSeqIdTemp*), and an average at 0.5 Å to the best possible template (*bestSeqIdTemp* and *bestStructTemp*). Finally, the addition of two more templates that are closer in sequence identity increase the quality of 0.3 Å in regards to theoretical best available approach (*3bestSeqIdTemp* and *bestStructTemp*, respectively).

**Figure A2:**
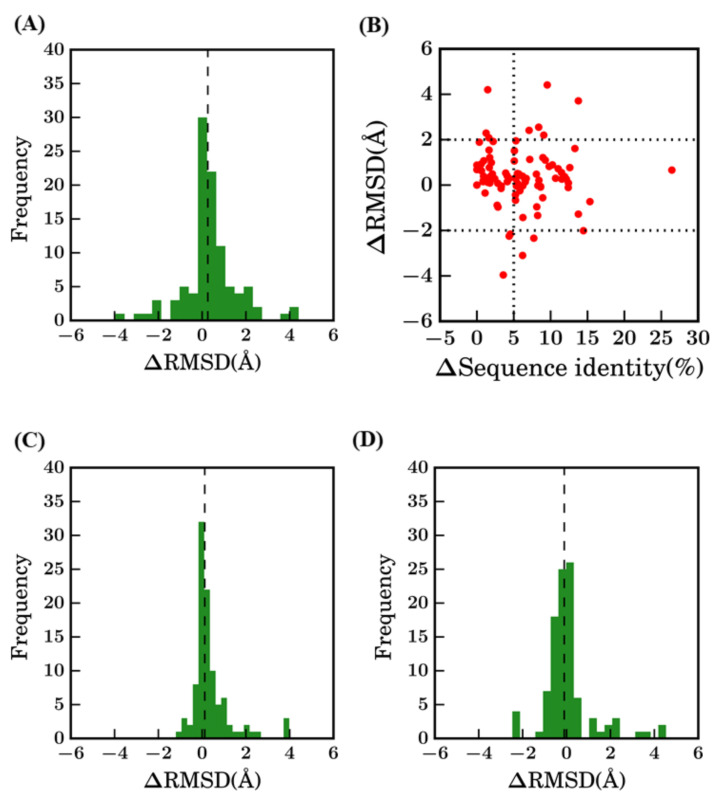
Difference between selected models. Difference between RMSD (ΔRMSD) of selected models (**A**) between *bestSeqIdTemp*—scenario 1 and *bestStructTemp*—scenario 2, (**C**) between scenario 1 and scenario 3—*3bestSeqIdTemp*, and (**D**) between scenario 2 and scenario 3. (**B**) ΔSequence identity (%) (between query and template sequences of scenario 1 and scenario 2) vs ΔRMSD (selected model with the original crystal structure).

However, comparison of these different results shows that scenario 1 is not always the most unfavorable method (see Figure A2). Indeed, 22% of the proteins have a better structural model using scenario 1 (*bestSeqIdTemp*) compared to scenario 2 (*bestStructTemp*, see Figure A2A). No correlation or clear trend can be observed from the comparison of the difference in terms of sequence identity to the difference in terms of structural similarity (see Figure A2B). When using multi-templates (scenario 3—*3bestSeqIdTemp*), the number of cases where best models have higher RMSD value are more limited, but still represent 12% of the models (see Figure A2C). The improvement of RMSD values is also slightly limited, it is better distributed when scenarios 2 and 3 are compared (see Figure A2D).

#### 2.2.2. Difficult Cases

The factors that mainly affect the quality of models are the sequence identity and structural similarity between the query and the template. Thus, to investigate their impacts, results are analyzed under two categories with models having an RMSD higher than 3 Å, or not. Table 1 summarizes cases that can be considered difficult with RMSD values higher than 3 Å for at least one scenario. For the 22 cases, nine are worst for scenario 1, three for scenario 3 and, more surprisingly, 10 for scenario 2. While the first cases (*bestSeqIdTemp*) are expected, the three examples of scenario 3 could imply multiple sequences (*3bestSeqIdTemp*) and could provide spurious constraints that decrease the quality of the structural models. Besides, for the worst scenario 2, the reasons seem more complex, as the closest structural template does not provide the best structural model: the small differences in CDRs are more important than expected. The best models are provided 11 times by scenario 2 and 11 times by scenario 3.

**Figure A3:**
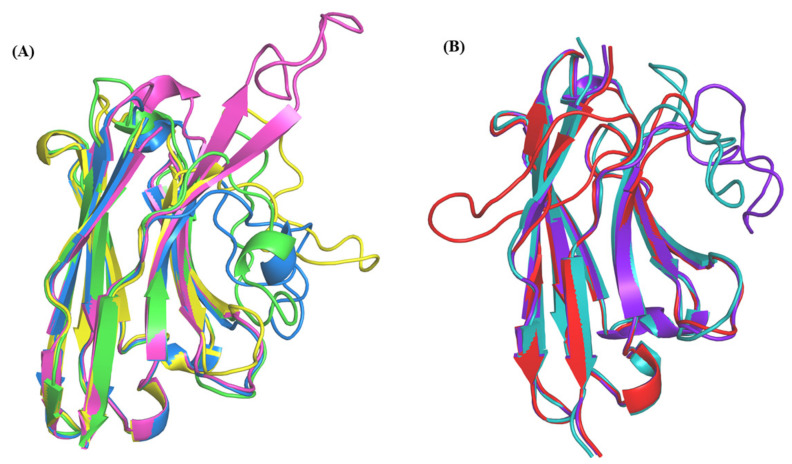
Representation of structural templates and models of query sequences from (**A**) PDB id 3G9A, (**B**) PDB ID 3SN6.

At first, the analyses of raw values do not provide any simple explanation. For example, V_H_Hs with PDB IDs 1KXV:D, 5GXB:B, and 3K81:B, all have templates with 63–69% sequence identity and the RMSD of the template with the structure is always between 1.4 and 2.0 Å, suggesting correct structural similarity. Other cases are also striking, the highest approximation value is observed for the structural model of a V_H_H complexed to green fluorescent protein (PDB ID 3G9A:B) leading to a large RMSD of more than 7.8 Å with scenario 1 (*bestSeqIdTemp*), while its structural template is “only” at 4.7 Å (See Figure A3A). The fact that this V_H_H is complex may not be the best explanation for the observed difference, as the most structurally similar V_H_H could have given a good structural approximation. This is not the case, as often outliers are difficult to capture.

The second most striking case is one V_H_H binding to a GPCR protein (namely PDB ID 3SN6:N, see Figure A3B); it has its best structural template that is solved as a complex with nucleoporin (PDB ID 5C3L:D, RMSD of 1.4 Å) and shares also the best sequence identity (SI of 83%). However, it only results in a 6.9 Å structural model. This poor quality result is mainly due the long CDR3 in 3SN6:N, which has 14 residues more compared to the template 5C3L:D. The CDR3 was slightly improved when two more templates were added with multi-template scenario 3 (*3bestSeqIdTemp*) as the structural template CDR3 was slightly longer with dive residues more than 5C3L:D, but still nine residues were without template information.

**Figure A4:**
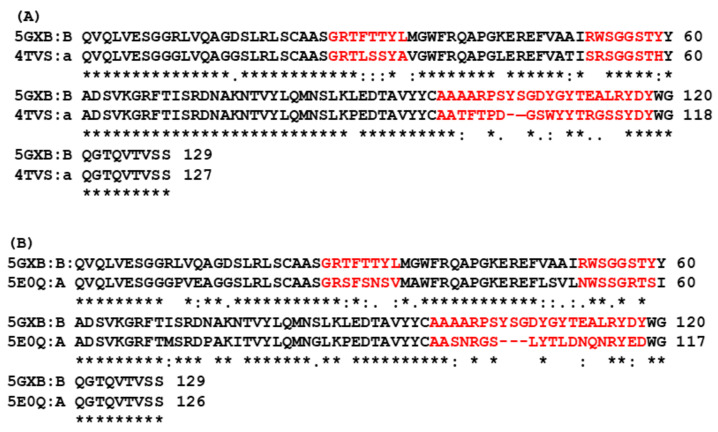
Sequence alignment of V_H_H binding to LacY (PDB ID 5GXB:B, 129 aa) with its templates. (**A**) Alignment with template PDB ID 4TVS:A (127aa) in *bestSeqIdTemp* scenario, (**B**) Alignment with template PDB ID 5E0Q:A (126 aa) in *bestStructTemp*. The highlighted regions in red in each alignment are CDRs separating the FRs. The “:” under aligned residues indicate strong similarity in amino acid conservation, “*” indicates identical residues and ‘.’ indicates weak conservation.

In fact, most of the alignments of these poor-quality models have more gaps in their alignment in comparison to the rest of the cases in a similar scenario (more than five gaps), 12 out of 14 alignments, 12 out of 12 (all alignments), and 7 out of 9 in scenarios 1, 2 and 3 respectively. To add further, in the case of a V_H_H binding to LacY (PDB ID 5GXB:B, 129 aa length) is particularly relevant. When modeled with scenario 1 template PDB ID 4TVS:A (SI 78%, see Figure A4A), this V_H_H has higher RMSD compared to scenario 2 template PDB ID 5E0Q (SI 69% of 126 aa, see Figure A4B), the former does not improve even after the addition of two more closely related templates (mean sequence identity (MSI) of 77%). These results are interesting as they underline cases for which there is: (i) with no significant difference in sequence length, and (ii) lower sequence identity, a template can lead to better structural models.

More cases in which the best models have RMSD < 3.0 Å in both scenario 1 and scenario 2 were examined. Using sequence length (especially in CDRs) as a criterion influencing their structural similarity, 18 of such cases where the sequence lengths between query and template sequences were less than 3 aa, and were found to be structurally dissimilar. As an example of these cases, a case of 5GXB:B modeling, scenario 2 was better than scenario 1, conveying using the best sequence identity template to build the best structural model must be used carefully with V_H_Hs.

#### 2.2.3. Impact of Sequence Similarity between Template and Query on Selected Models

**Figure A5:**
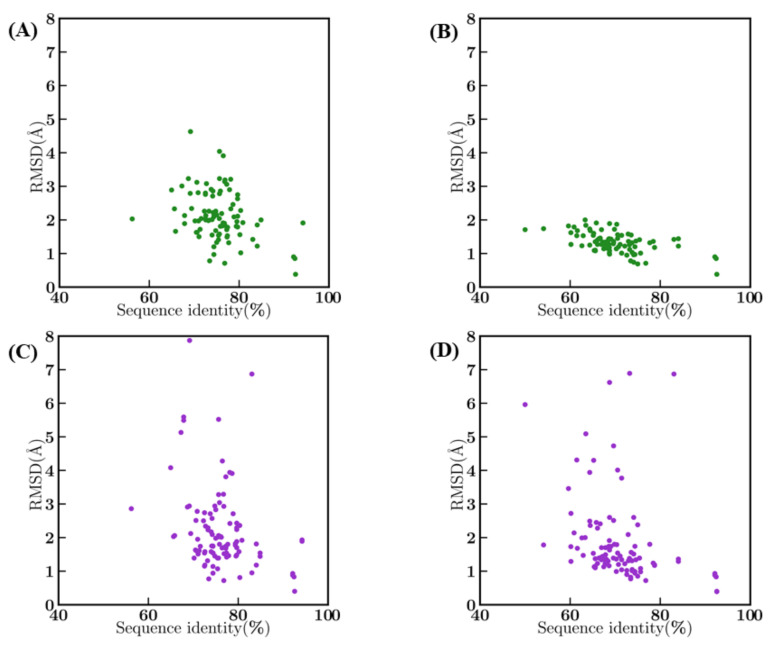
Sequence identity *vs.* RMSD. Between query and template *crystal* structures (**A**) in *bestSeqIdTemp* and (**B**) in *bestStructTemp*, and between best model and original crystal structures (**C**) in *bestSeqIdTemp* and (**D**) in *bestStructTemp*.

An analysis of the systematic impact of sequence identity between the template and the query sequences on structural model qualities measured by RMSD were examined in detail. As expected, *bestSeqIdTemp* showed a cluster between 70 and 80% sequence identity and 1–3 Å RMSD, with no direct correlation between sequence identity and RMSD (see Figure A5A). Interestingly, some templates are already at more than 4 Å from the query structure. For *bestStructTemp* cases, the sequence identity of the query sequence with the template in this scenario is lower (as we have a priori knowledge of structures of the query sequences), they have a better structural approximation, forming a cluster between 60 and 80% and 1–2 Å RMSD. Here, no correlation could be observed between sequence identity and RMSD (see Figure A5B). The generation of structural models is directly dependent of the structural proximity with the query, but is not the only parameter that impacts the quality of the model. It is not shocking to see that for both *bestSeqIdTemp* and *bestStructTemp* scenarios (see Figure A5C,D), the best structural models have higher RMSD with the true structures. The *bestStructTemp* scenario modes shows more deviation in RMSD in comparison to crystal structures than models from the *bestSeqIdTemp* scenario, leading to eight structural models at more than 4 Å RMSD when the RMSD is, at most, less than 2 Å with the query structure.

**Figure A6:**
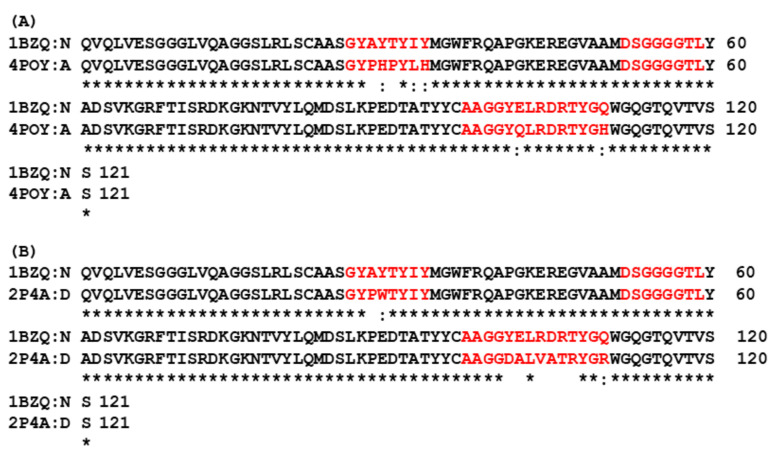
Sequence alignment of V_H_H complexed with RNase I (PDB ID 1BZQ:N) with templates. (**A**) Alignment with template PDB ID 4POY:A in bestSeqId*Temp* scenario, and (**B**) alignment with template PDB ID 2P4A:D in bestStructTemp scenario. The highlighted regions in red in each alignment are the CDRs separating the FRs. The “:” under aligned residues indicate strong similarity in amino acid conservation, “*” indicates identical residues.

However, even for query and template sequences of the same residue lengths and of high sequence identity, the RMSD can vary. For example, the V_H_H domain from 1BZQ:N is modeled with (i) 4POY:A (94% sequence identity) for scenario 1; (ii) 2P4A:D (92% sequence identity) for scenario 2. They both share equal lengths to the query and have RMSDs of 1.9 Å and 0.4 Å, respectively. Even with this strikingly minimal difference (only 2% sequence identity, in CDR 1 and CDR3 in both cases) between the two template sequences), the induced structural change is three times greater in terms of RMSD values of models from both cases. In fact, there are more residues conserved in the alignment of the query (i.e., 1BZQ:N) with the template from scenario 1 (i.e., 4POY:A) than for the template from scenario 2 (i.e., 2P4A:D), which, in the CDR regions, are contributing to the change in RMSD (see Figure A6). 

**Figure A7:**
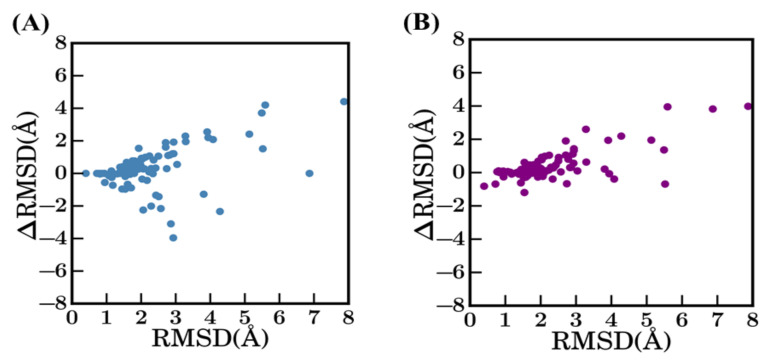
Comparison of scenario 1—*bestSeqIdTemp* with other scenarios. The RMSD of best models in Scenario 1 (*x*-axis) vs. (**A**) the difference in terms of RMSD with selected models of scenario 2 and (**B**) of scenario 3 (*y*-axis).

Structural similarity between best models of different scenarios (see Figure A7A,B) suggest that (a) templates in scenario 2 had a more negative impact than scenario 1 and (b) as the template used in scenario 1 is also used in scenario 3, along with two more templates, the structural similarity is not too divergent in most regions of the V_H_H. Indeed, there are surprising cases where the best structural template had more deviation than the best sequence identity template. Comparison of scenarios 1 and 3 (see Figure A7B) shows that no such drastic deviations were observed. Nonetheless using the best possible structural template was able to reduce the worse RMSD in scenario 1, around 8 Å to 4 Å. Indeed, multiple sequence alignments properly done might have better structural approximation.

### 2.3. Assessment of Structural Models in FRs and CDRs

**Figure 4:**
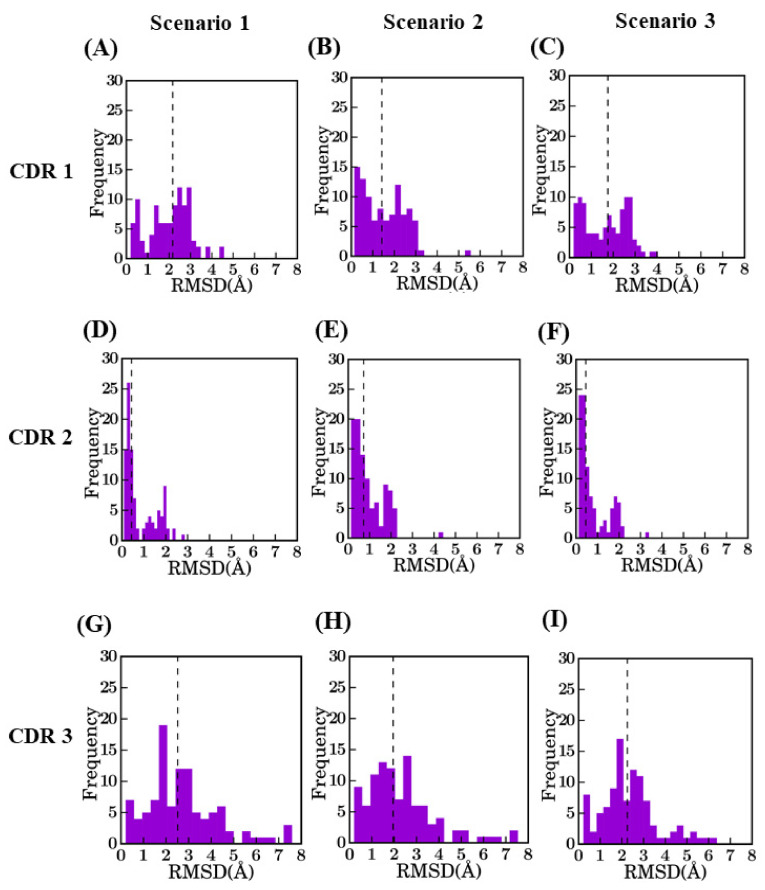
Distribution of RMSD values for different CDRs between selected model and crystal structure. CDR1 for (**A**) *bestSeqIdTemp* (median value of 2.2 Å), (**B**) *bestStructTemp* (1.4 Å), and (**C**) *3bestSeqIdTemp* (1.8 Å), CDR2 for (**D**) *bestSeqIdTemp* (0.4 Å), (**E**) *bestStructTemp* (0.7 Å), and (**F**) *3bestSeqIdTemp* (0.5 Å), CDR3 for (**G**) *bestSeqIdTemp* (2.5 Å), (**H**) *bestStructTemp* (1.9 Å), and (**I**) *3bestSeqIdTemp* (2.4 Å).

**Figure A8:**
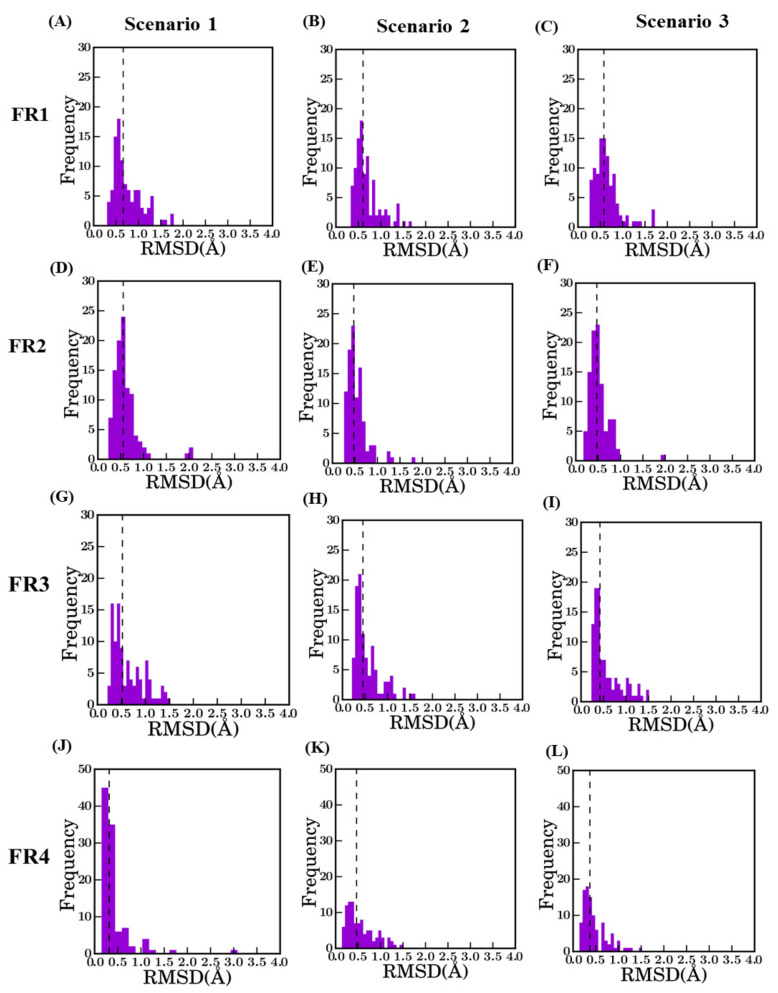
Distribution of RMSD in FR between best model and crystal structure. The dotted line represents the median value in each plot; they are indicated in brackets. FR1 (**A**–**C**), FR2 (**D**–**F**), FR3 (**G**–**I**), and FR4 (**J**–**L**) with scenario 1 (**A**: 0.66 Å, **D**: 0.56 Å, **G**: 0.52 Å, and **J**: 0.32 Å), scenario 2 (**B**: 0.61 Å, **E**: 0.4 Å, **H** 0.45 Å and **K** 0.47 Å) and scenario 3 (**C**: 0.58 Å, **F**: 0.47 Å, **I**: 0.42 Å, and **L**: 0.37 Å).

After analyzing complete V_H_H best models, region-wise analyses were undertaken to understand the precise differences in them (see Figure 4 for CDRs and Figure A8 for FRs). FRs are not as simple as anticipated. Despite that, the RMSD values are limited on average, two points can be noted: (i) for every scenario and every type of FRs, some RMSD values are higher than 1 Å. It is surprising since FRs are supposed to be the most rigid part of the V_H_H to handle; (ii) scenario 2 is only better for FR2 (0.4 Å), while scenario 3 is better for FR1 (0.6 Å) and FR3 (0.4 Å) and even scenario 1 for the short FR4 (0.3 Å). It underlines a specific difficulty of V_H_H topology; high local sequence identity with a lot of β-sheet does not mean easy modeling.

The RMSD in CDR regions is of much higher range, especially for CDR3. The CDR2 region has the lowest RMSD values, as it is the smallest in length. It is unexpected to have high RMSD values for CDR2 (only two cases can be observed are more than 2.0A). Modeling of CDR1 is more complex with RMSD values at best are close to 1.5 Å for scenario 2. CDR3 has the maximum diversity owing to changes in length and sequence identity. The addition of a multi-template does not greatly improve the structural approximation (gain of 0.27 Å) and is close to the theoretical limit of RMSD for scenario 2 (which is at 0.35 Å).

### 2.4. Conservation of CDR Loop Termini Distances in Best Models in Comparison to Crystal Structures

**Figure 5:**
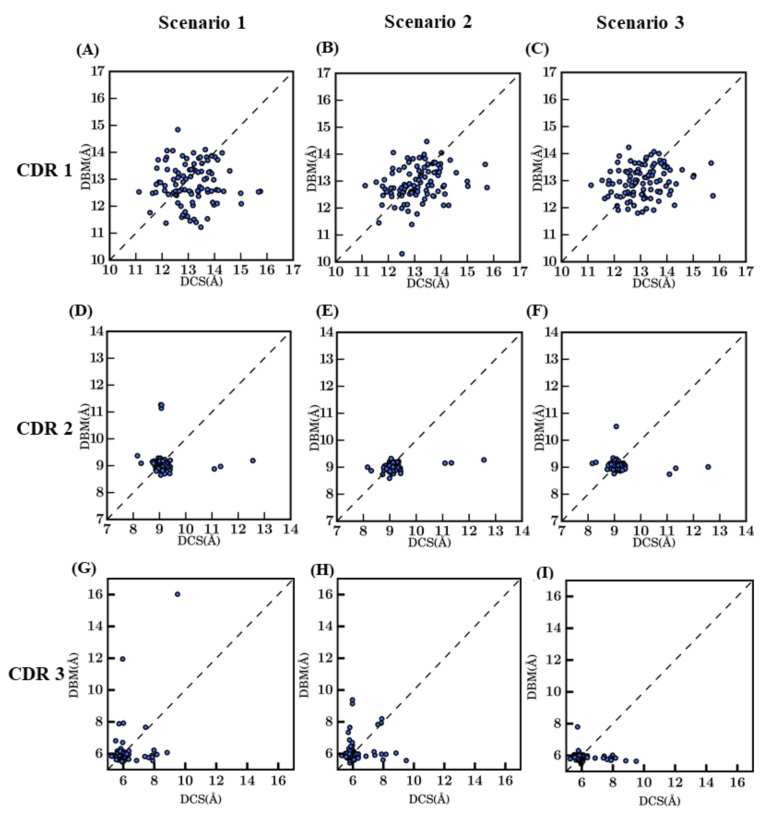
Distance between the Cα residues of CDR termini in crystal structures and corresponding selected models. (**A**) to (**C**) CDR1, (**D**–**F**) CDR2, and (**G**,**H**) CDR3, scenario 1—*bestSeqIdTemp*, corresponds to (**A**,**D**,**G**), scenario 2—*bestStructTemp* to (**B**,**E**,**H**), while scenario 3—*3bestSeqIdTemp* is (**C**,**F**,**I**). The *x*-axis values represent the distance observed in crystal structure (DCS (Å)) and the *y*-axis values the distance observed in the best model (DBM (Å)).

Prediction of loop conformations is known to depend on the conformations of the anchor residues and the distance between the anchor residues [29,30,31,32]. To understand if the distances between the loop termini in the templates influence the distances in the models, these distances between Cα atoms of the CDR loop termini were computed. Figure 5 shows the comparison of loop termini distances in query crystal structure versus their corresponding best model in each scenario. Figure 5D–I shows that the Cα distances of CDR2 and CDR3 termini are well approximated with few deviations compared to the native structures. Hence, distances for CDR2 are at less than 1 Å between crystal structures and models, with only 7 models are at more than 1 Å (with 2 are more than 4 Å). Interestingly, it is found for every scenario. For CDR3, it is the same trend, but it is accentuated for scenario 1, which is supposed to be the best approach. The CDR1 distance comparison showed larger deviations in most of the loops modeled. On average, CDR1 distance is of 13 Å, but the predicted distance is at +/− 1.5 Å, for every scenario, sampling with no preference; thus, adding a new difficulty in the V_H_H model prediction (see Figure 5A–C). This loop is particularly interesting as it extends between the two β-sheets of the domain and is the largest distance of the three loop termini.

### 2.5. Case Studies: Analyzing Local Backbone Conformations of Two V_H_H Domains in Different Scenarios for Two Representative Modeling Case Studies of V_H_H

The first is a llama V_H_H targeting muscarinic acetylcholine receptor M2 (PDB ID 4MQS:B, 125 residues) [33], while the second is from dromedary and binds Phage Tuc2009 Baseplate Tripod (PDB ID 5E7B:A, 131 residues with additional disulfide bridge) [34]. These two were chosen for the following reasons: (i) the structural models obtained by the three scenarios for the first one has interesting structural similarity, and (ii) structural models of the second one show greater deviations.

#### 2.5.1. Case 1

**Figure 6:**
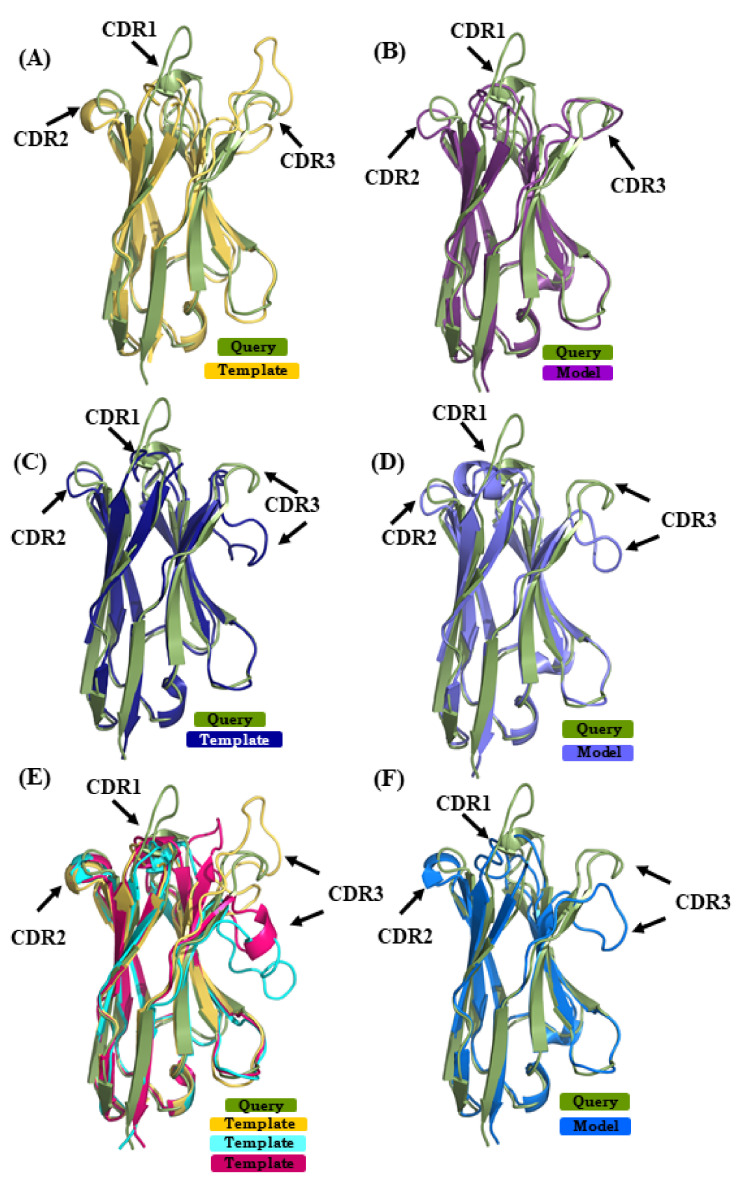
Superimposition of V_H_H query structure (V_H_H binding to human M2 muscarinic acetylcholine receptor, PDB ID 4MQS:B) and templates/models. The query PDB ID 4MQS:B [33] is superimposed with templates used in (**A**) scenario 1 (in yellow PDB ID 5HDO:A [35], (**C**) in scenario 2 (in dark blue PDB ID 3STB:B [36], and (**E**) in addition to template from scenario 1, PDB ID 4TVS:a [37] in cyan, and in pink PDB ID 4LAJ:H [38]. (**B**,**D**,**F**) are best-selected models using templates shown in (**A**) (in purple), (**C**) (in magenta), and (**E**) (in light blue).

The query sequence from V_H_H 4MQS:B was modeled with structural template 5HDO:A [35] (sequence identity or SI of 72%) in case of scenario 1, 3STB:B [36] (SI 64%) in case of scenario 2 and in case of scenario 3, combining 4TVS:a [37] and 4LAJ:H [38] along with 5HDO:A (mean sequence identity or MSI 70%). The best models from the respective scenarios 1, 2, and 3 had RMSD values of 2.3 Å, 2.5 Å, and 2.7 Å to the original structure. Figure 6 shows the superimposition of the query structure and the different structural template/best model. The β-strand structural similarity is excellent in all of the cases. Figure 6A,C,E show the superimposition of the 4MQS:B and its template in each scenario. It is obvious that CDRs 1 and 3 of 4MQS:B (olive green color) display no similarity with any of the templates. The selected models of scenarios 1, 2, and 3 are shown in Figure 6B,D,F. CDR1 and CDR3 loops that are structurally dissimilar in all the templates also remain quite dissimilar in these selected models.

**Figure A9:**
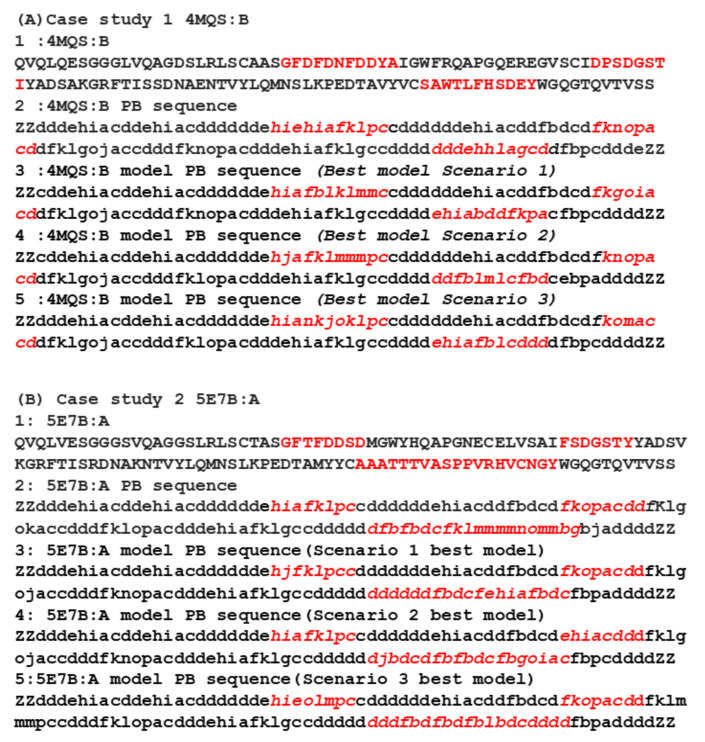
Amino acid and PB sequences of two V**_H_**Hs (PDB IDs 4MQS:B and 5E7B:A). (**A**) In case study 1; 1: PDB ID 4MQS:B amino acid sequence; 2: PB sequence of PDB ID 4MQS:B crystal structure, 3, 4, and 5 PB sequences of models from *bestSeqIdTemp*, *bestStructTemp,* and *3bestSeqIdTemp* scenarios. (**B**) In case study 2; 1: PDB ID 5E7B:A amino acid sequence, 2: PB sequence of PDB ID 5E7B crystal structure, 3, 4, and 5 are PB sequences of models from *bestSeqIdTemp*, *bestStructTemp,* and *3bestSeqIdTemp* scenarios. The highlighted regions in red are the CDRs.

To add further, native structures, structural templates and selected models have been analyzed using protein blocks (see Figure A9A) [39]. The CDR1 sequence **‘GFDFDNFDDYA’** adopts a ***‘hiehiafklpc’*** PB signature in the structure, which none of the models exhibit. Although all three of them exhibit more or less similar PB sequences at the start of the loop, ***hiafblklmmc,hjafklmmmpc, hiankjoklpc,*** they quickly deviate into highly divergent signatures. The CDR2 sequence **‘DPSDGST’** shows ***‘fknopacd’*** PB signature in the crystal structure, which, in the best models, are slightly changed to ***‘fkgoiacd’, ‘fknopacd’*** and ***‘fkomaccd’***. These results are accurate and highly close in terms of PBs (exact match for scenario 2, two mismatches only for the two other scenarios). 

It seems that the amino acids aspartate and proline at the start of the group and provide tight constraints in all of the modeling cases. In the case of CDR3, the sequence **SAWTLFHSDEY** shows PB signature ***‘dddehhlagcd’*** in the crystal structure, which is largely modified in ***ehiabddfkpa, ‘ddfblmlcfbd’,*** and ***‘ehiafblcddd’*** in scenarios 1, 2, and 3. These conformations are far away from the V_H_H crystal structure, with only a few PBs *d* at the extremity for scenario 2 and no common PBs for scenario 1 and 1 for scenario 3. This example underlines that although models are closely similar in terms of RMSD, their CDRs can be quite different in conformations.

#### 2.5.2. Case 2

**Figure 7:**
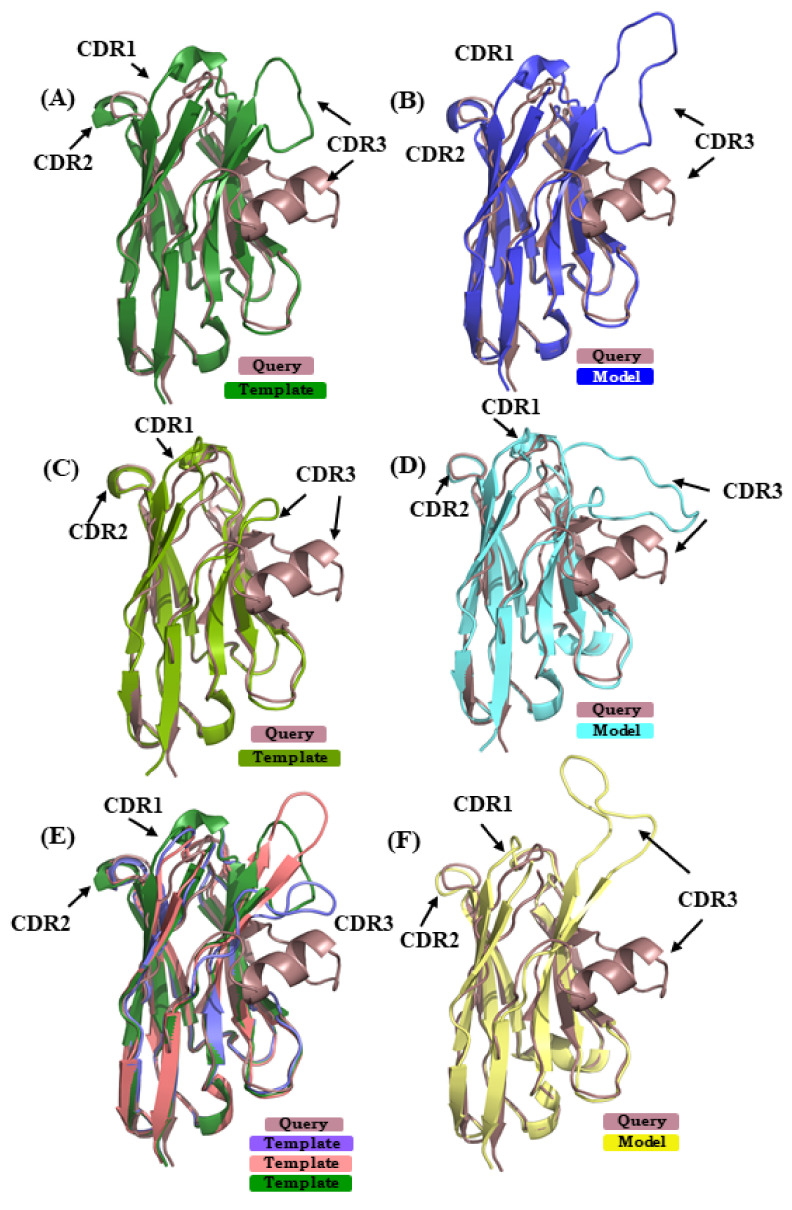
Superimposition of query structure V_H_H binding to phage Tuc2009 receptor binding protein (PDB ID 5E7B:A) with templates and models. The query structure V_H_H that was binding to phage Tuc2009 receptor binding protein (PDB ID 5E7B:A [34] is superimposed to different templates/models: (**A**), used in scenario 1 (PDB ID 1SJX:A [40]; (**B**) in scenario 2 (PDB ID 5H8D:A [41]); and (**C**) in addition to 1SJX:A, PDB ID 4ZG1:F [42], PDB ID 5JQH:C [43] are shown, respectively. (**B**,**D**,**F**) are the best-selected models using templates shown in (**A**,**C**,**E**).

In case study 2, the V_H_H sequence 5E7B:A [34] is modeled using (i) 1SJX:A [40] (SI 74%) in scenario 1, (ii) 5H8D:A [41] (SI 70%) in scenario 2, and (iii) in case of scenario 3 4ZG1:F [42] and 5JQH:C [43] were additional used, along with the template from scenario 1 (mean SI 74%). The RMSD of the best model from scenarios 1, 2, and 3 are 5.5 Å, 4.0 Å, and 6.2 Å, respectively. Figure 7A,C,E show the superimposed representations of the query structure and the templates used. It is obvious that none of the template structures can provide a good model in the region of CDR3; this case is highly related to the one observed in [28]. Figure 7B,D,F show the best model in scenarios 1, 2, and 3, in each case superimposed to 5E7B:A crystal structure. As seen in the previous case study of 4MQS:B, PBs were assigned (see Figure A9B). For CDR1, the amino acids sequence **‘GFTFDDSD’** is associated to PB sequence ***hiafklpc,*** while in its models it is associated to ***hjfklpcc, hiafklpc,*** and ***hieolmpc*** for CDR1 from scenarios 1, 2, and 3, respectively. Hence, differences in their PB signature are seen in scenario 1, as only the first and last PBs are in common, and in scenario 3, as only the two first and two last PBs are in common; CDR1 PB signature in model from scenario 2 is exactly the same. For CDR2, the amino acids sequence **‘FSDGSTY’** is associated to the PB signature ***‘fkopacd’***. The models provided PB series ***fkopacd, ehiacdd,*** and ***fkopacd*** in scenarios 1, 2, and 3, respectively. The CDR2 signatures from scenario 1 and scenario 3 are identical to the crystal structure signature. In case of CDR3 sequence **‘AAATTTVASPPVRHVCNGY’** shows PB signature ***‘dfbfbdcfklmmmmnommb’,*** which has extended conformations in the beginning (presence of PBs *d*, *b* and *f*) and helical (PB *m*) in the end. In the best models selected from the three scenarios, the CDR3 is assigned the PB signatures ***ddddddfbdcfehiafbdc*** (scenario 1)***, djbdcdfbfbdcfbgoiac*** (scenario 2)*, **dddfbdfbdfblbdcdddd*** (scenario 3)**.** It can be inferred that none of these model CDR3 PB signatures are close to the crystal structure PB signature. Moreover, they tend to be more extended in their conformation than helical in all cases. This example shows that most of the regions can be modeled with good accuracy, while CDR3 is more difficult to model and dictate the high global RMSD seen here. 

#### 2.5.3. Assessment of Backbone Conformational Sampling in Models Using Protein Blocks

In previous sections, we have underlined the interest of specific approaches by selecting a ‘best’ model. The generation of the large number of models can also provide interesting information. Hence, to understand how each modeling scenario is different from the other and what conformations each of them have sampled is carried out by analyzing PB signatures of both case studies in each scenario using PB maps. The two examples presented in Figure 6 and Figure 7 were chosen.

**Figure A10:**
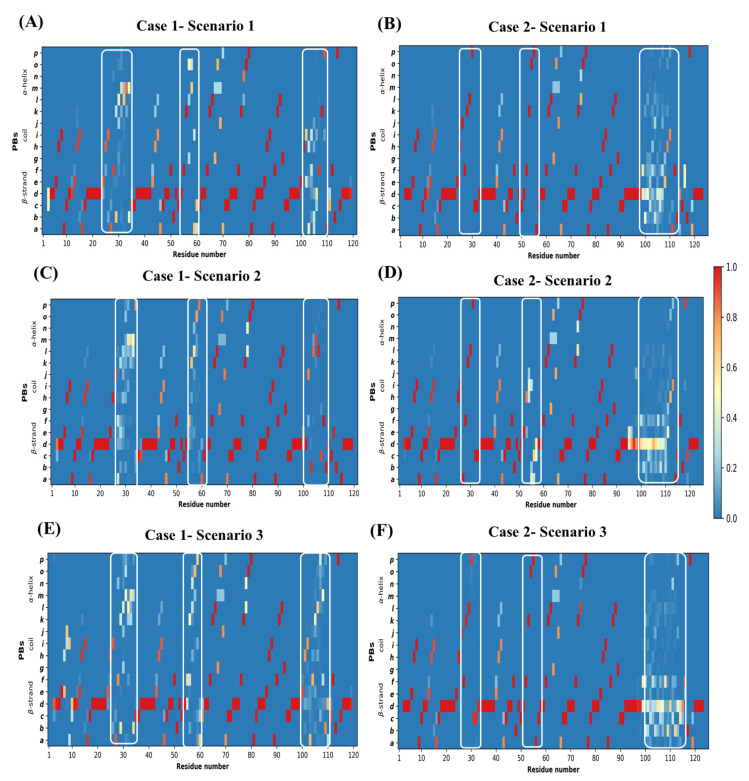
Conformational Sampling in models visualized using protein blocks. Conformational diversity in models represented as PB maps. (**A**,**C**,**E**) are PB maps for query sequence PDB ID 4MQS:B modeled in scenario 1, 2, and 3, respectively. (**B**,**D**,**F**) are for query sequence PDB ID 5E7B:A modeled in scenario 1, 2, and 3, respectively. The regions enclosed by white rectangles are CDR1, CDR2, and CDR3, denoted from left to right in each figure.

For 4MQS, little diversity is observed for FRs, conformational sampling is limited but can be impacted differently in each scenario (see Figure A10A,C,E). Hence, FR1 is well conserved in scenarios 1 and 2 modelling, but shows some variations for scenario 3; all other FRs are equivalent in all scenarios. Conformational diversity in CDR 1 (positions 26–36) and CDR 2 (55–62) and especially in CDR3 (97–110) is significantly more pronounced. However, in case of CDR3, the strong PB signature in scenario 1 and scenario 2 is not at all preserved in scenario 3. Hence, the multi-template allows the deepest conformational exploration.

For 5E7B:A, models have sampled strand conformation for CDR3 in all the modeling scenarios (see Figure A10B,D,F). There are very few positions for this case in scenarios 1 and 3 that show conformational diversity in CDR1 and CDR2. The FRs also show less diversity in all cases except for just after CDR2 (position 63–68).

**Figure 8:**
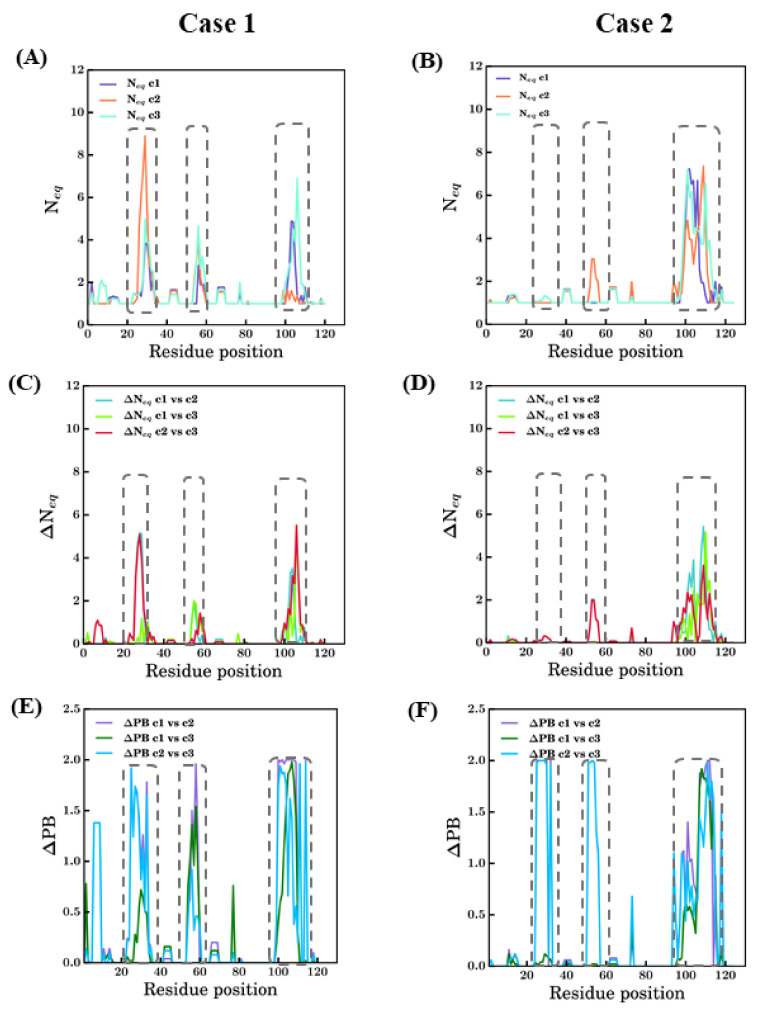
Summary of changes in conformational sampling. Two modeling case studies of PDB ID 4MQS:B (Case 1) and PDB ID 5E7B:A (Case 2) are presented. (**A**,**B**) PB entropy (*N*_eq_) [22] (raw values can be found in Supplementary Files S1 and S2); (**C**,**D**) change in PB entropy (Δ*N*_eq_) [44] and (**E**,**F**) differential PB signature at each position (ΔPB) in different scenarios [44,45]. Each graph has all the three scenarios represented in different colors and notations c1, c2, and c3 for scenarios *bestSeqIdTemp*, *bestStructTemp,* and *3bestSeqIdTemp*. The dotted rectangles approximately denote the regions CDR1, CDR2, and CDR3 indicated from left to right in each figure.

PB entropies (*N*_eq_ [22]) are shown in Figure 8. PB *N*_eq_ is able to analyze and capture information of conformational diversity. For 4MQS:B (see Figure 8A,B), the CDRs have *N*_eq_ values higher than 2. CDR1 showed greater values of *N*_eq_ in scenario 2 (more than 8) followed by scenarios 3 and 1. Whereas for CDR2 and CDR3, scenario 3 shows high and higher values of ~5 and ~7, respectively; both are considered high, suggesting a lot of diverse sampling. Many FRs along with CDRs, positions 26–36, 55–62, 97–110, have *N*_eq_ values are >1, suggesting some limited sampling of local conformation, e.g., *N*_eq_ of 2 for scenario 3 is seen four times. The differences in the conformations advocate the influence of template through the constraints they provide to the model-building algorithm.

Analysis of 5E7B:A models in terms of *N*_eq_ shows drastic differences in comparison to 4MQS:B models. The CDR3 region (93–112) models from all three scenarios show high *N*_eq_ values, more than 6 in this region. For CDR1 region (26–33 aa region), no scenarios exhibit much conformational diversity (see Figure 8B). It is surprising as the CDR1 region is the next difficult region to model. In the CDR2 region (52–58), except models from scenario 2, which show slightly higher *N*_eq_, the other scenarios are conformationally less diverse. Even in the FR regions, conformational diversity is less observed compared to the previous case study.

A quantitative comparison of *N*_eq_ values between any two scenarios is calculated and represented as ΔN_eq_ values (see Figure 8C,D). It mainly underlines a large difference for 4MQS in CDR3 between all scenarios and in CDR1 between scenarios 2 and 3; for 5E7B, it is only distinctly different in CDR3 regions while the rest of the modelled regions in all the scenarios do not show much variations.

To apprehend more precise, qualitative differences, in terms of PBs, the ΔPB is computed [44]. In addition to Δ*N*_eq_ [44], ΔPB is used to compare two different scenarios, but it gives qualitative information. Its value ranges from 0 to 2, suggesting identical conformations at 0 and completely different conformations at 2. The comparison of ΔPB values for scenario 1 vs. scenario 2, scenario 1 vs. scenario 3, and scenario 2 vs. scenario 3 are shown for case studies in Figure 8C and Figure 7D. In Figure 8C, the three CDR regions (positions 25–33, 52–58, and 97–110) show ΔPB values for some cases around 2. This analysis underlines local protein conformations that are significantly different, e.g., the end of CDR2 regions between all scenarios. However, the FR regions, which should be more tightly constrained, also show non-zero ΔPB values in the regions of FR1 between scenarios 2 and 3, after CDR1 (42–45), after CDR2 (62–65), and for positions 74–76, mostly in scenario 1 vs. scenario 3 and scenario 2 vs. scenario 3. In case of 5E7B:A, similar to 4MQS:B, the CDR3 region (93–112) shows much diversity in all comparisons as represented by the ΔPB values. The ΔPB values for scenario 2 vs scenario 3 are close to 2 in the CDR 1 (26–33) and CDR 2 (52–58). These regions show local conformations entirely different between the two scenarios. Some regions other than CDR have non-zero ΔPB values in all comparisons; amongst these, one region stands slightly conspicuous, the amino acid region 74–76 for all scenarios. The antibody research community considers this region as the fourth CDR loop, in both cases 5E7B:A and 4MQS:B this appears to have non-zero ΔPB values in this region, suggesting conformational diversity is possible in this region. Thus, this region might not confer many constraints as a regular secondary structured region, which results in higher sampling in this region. The above analysis of modeling in different scenarios shows the applicability of PBs as a tool for analyzing conformational sampling during modeling and its efficiency in discriminating local conformations.

### 2.6. Systematic Modeling of V_H_H Domains (Scenario 4—All)

**Figure 9:**
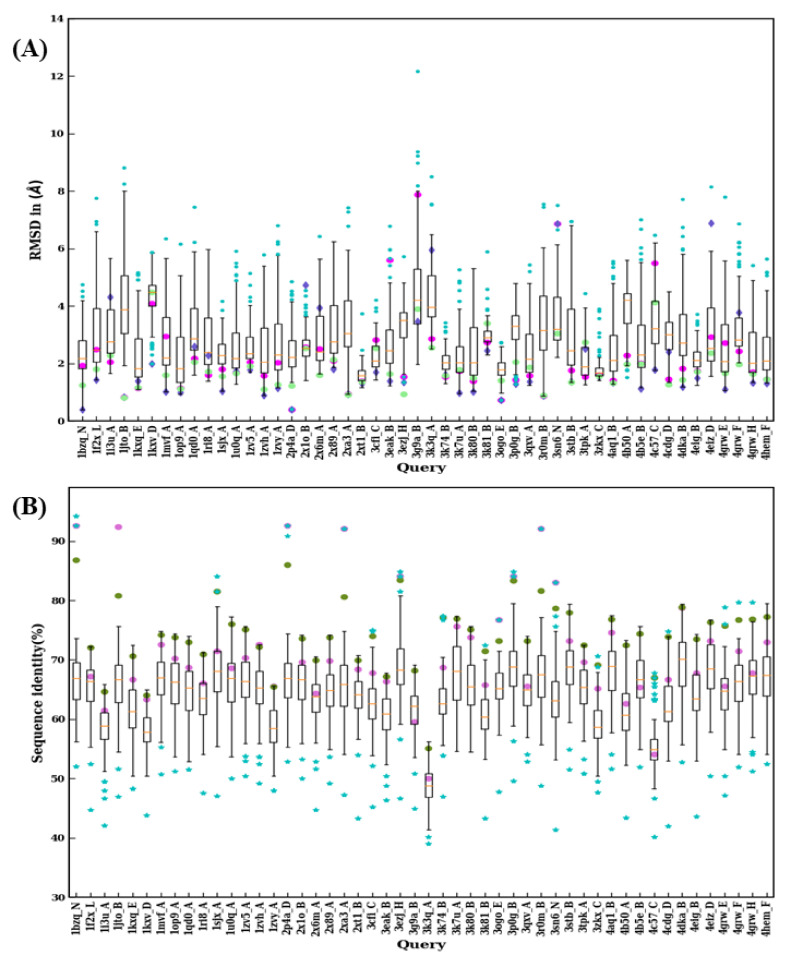
Structural similarity and sequence identity for first 50 V_H_H. (**A**) RMSD of best models from 99 modeling cases computed against the crystal structure. The RMSDs of best models from scenario 1, 2, and 3 are marked in magenta, purple, and green, respectively. (**B**) The values of sequence identities are shown. The sequence used in scenario 1 is in cyan at the upper limit, the one for scenario 2 is represented by a pink circle, and an olive green circle represents the average of the sequence identities, of the templates used for scenario 3.

**Figure 10:**
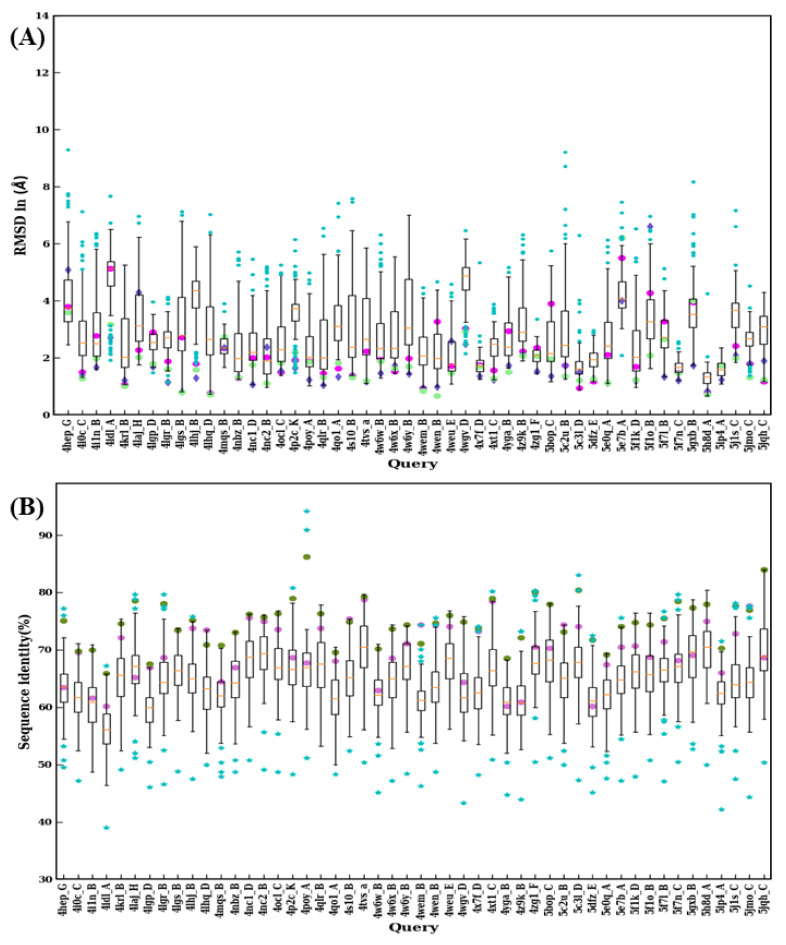
Structural similarity and sequence identity for last 50 V_H_Hs. (**A**) RMSD of best models from 99 modeling cases computed against the crystal structure. The RMSDs of best models from scenario 1, 2, and 3 are marked in magenta, purple, and green, respectively. (**B**) The values of sequence identities are shown. The sequence used in scenario 1 is in cyan at the upper limit, the one for scenario 2 is represented by a pink circle, and an olive green circle represents the average of the sequence identities, of the templates used for scenario 3.

Some examples presented in this study suggest that the choice of the template with the higher sequence identity is not always a guarantee to obtain the most structurally accurate model possible. Thus, to investigate whether it is the case for all V_H_H domains, each domain was modeled using the 99 remaining domains. A summary of the RMSD distribution of best model superimposed over the original crystal structure obtained for each domain is provided in Figure 9A and Figure 10A, as well as their corresponding sequence identities between the crystal structure and template used (see Figure 9B and Figure 10B). The pair-wise sequence identity distributions only had two outlier values for 3K3Q:A and 4IDL:A, which were lower than 40%. 1BZQ:N, 2P4A:D, 2XA3:A, 3R0M:B, and 4POY:A have pairwise sequence identity outliers above 90%. Most of the pairwise sequence identity values are between 50 and 80%, suggesting that it is the case for homology modeling.

The RMSD value distributions of 2XT1:B, 3K74:B, 3ZKX:C, 4X7F:D, 5F7N:C, 5H8D:A, and 5IP4:A show very narrow distribution because of smaller sequence lengths (≤115) suggesting a reduced structural misalignment due to CDR lengths (see Figure 9A and Figure 10A). Another notable observation is that when distributions were analyzed there were 30 cases where a lower RMSD value compared to any of the previous scenarios (such as best sequence identity, closest structural approximate, and multiple templates, see Figure 9B and Figure 10B) was observed. This observation suggests that there are other template features that could lead to a lower RMSD value. 

## 3. Discussion

To understand and appreciate the effects of the template backbone conformations on the models generated, a large-scale analysis of the V_H_H domain modeling was performed in this study. Four different scenarios were conceived, which cover most aspects of variable domain modeling. All of these scenarios were carried out with simple approaches, easily reproducible by the scientific community. Preliminary analyzes did not show significant gains with more advanced settings of Modeller.

The first scenario is the classical case, which is often used in V_H_H structure prediction studies and must be considered as the basic expectation. The main difficulty of this strategy is the length of CDR3 loops, and the conformation of the CDR1 loop, even if its length is, in most cases, constant. Moreover, 14% of the cases show an RMSD of the selected model higher than 3 Å. 

The second scenario reflects the best theoretical values possible, where the structural similarity, overall, plays a major role, and must be considered as the best theoretical result. Even if the CDR lengths do not match, the FRs in this scenario are expected to be highly similar between the templates and the query. In most cases, the structural similar template had the sequence length almost the same as the query, but for some cases, the differences in length of the region to be modeled, and the template, were drastically different. This, in particular, allowed CDR3 and a couple of CDR1 regions to adopt non-template dependent conformations. Hence, 12% of these V_H_Hs have a RMSD higher than 3 Å.

In the third scenario, the additional constraints are applied by adding two other templates with the next best sequence identity values. It leads to only 9% of models with RMSD values more than 3 Å. When RMSD of the best models were compared, only 22 cases in scenario 1 had better RMSD when compared to scenario 2, and this number was slightly reduced to around 12 cases when compared to scenario 3. The rationale here is simply: (i) best sequence identity template provides better constraints, or, (ii) adding more constraints at each residue position by adding more templates will improve the approximation.

**Figure A11:**
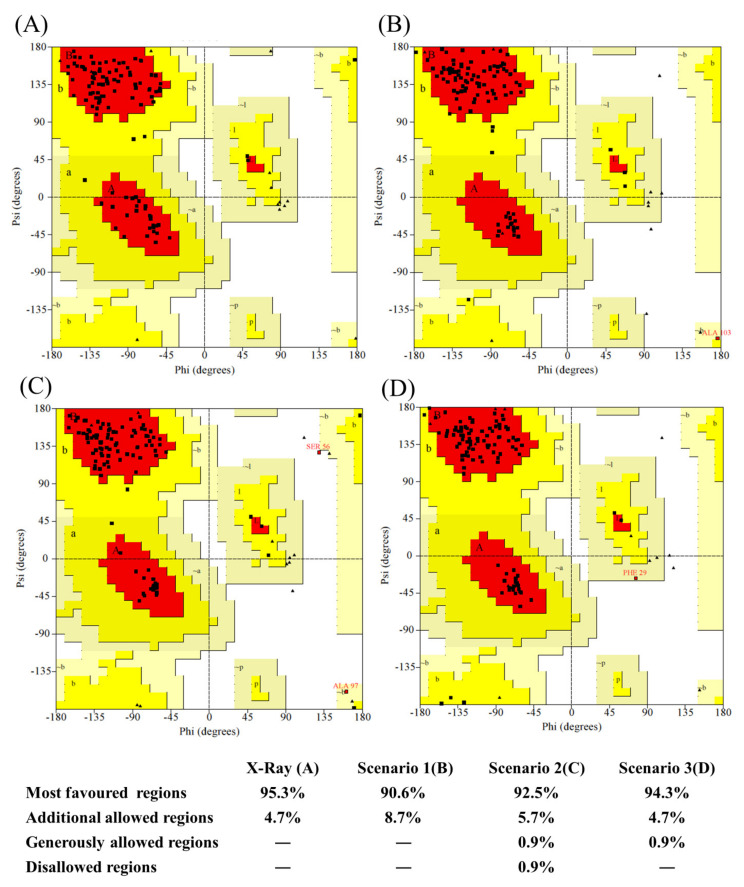
Ramachandran map distribution of residues. (**A**) 5E7B:A crystal structure, (**B**) best model from scenario 1 (*bestseqIdTemp*), (**C**) best model from scenario 2 (*beststructTemp*), and (**D**) best model from scenario 3 (*3bestseqIdTemp*).

Different regions (FRs and CDRs) of models from three scenarios were assessed using RMSD. As expected, CDRs showed higher values than the FRs; however, even in FRs, RMSD analyses from different scenarios, unexpectedly, scenario 2 showed higher values of median RMSD than the other two. Similarly, the distance between the Cα of residues in CDR loop termini between original crystal structures and their corresponding models were not preserved in every case. CDR1 had the highest distance between the termini and it appeared to change without any specific trend. The deviations at CDR2 and CDR3 loop termini were highly limited as their loops connected two adjacent β-strands where it was not the case for CDR1. This is an opportunity to think of a larger sampling than expected in terms of both loops and distance. It should be noted that the different scenarios always produce relevant 3D models, i.e., having no aberrant conformations. For example, the number of residues proposed in unfavorable regions is, on average 1%, and is always over 90% in the highly favorable regions of the Ramachandran map (see Figure A11). On average, the models selected have a better number of residues in the favorable regions of the Ramachandran map than the experimental structures [46].

Two examples were selected to explain to the reader the differences in the models influenced by the templates. The case study of V_H_H 4MQS:B revealed that even though the models are close in terms of RMSD, they could be quite different in local conformations. In the case of V_H_H 5E7B:A, it surprising to note that most of the protein, except the CDR3, could be modeled very efficiently, even from different templates. Further, the last scenario is studied if a better RMSD is possible with any other template other than best sequence identity template; in 30 cases, it was observed to have a model with lower RMSD than the other three scenarios. 

In our analyses, we find that FRs are simpler to understand than CDRs, but they have a direct effect on the quality of the models. Next, CDR1 has unexpected features; they are not very diverse in terms of conformations (and lengths), but the distances between loop extremities are not conserved and show variations between templates and models, irrespective of the scenario, while this is not the case for CDR2 and CDR3. Analyses of multiple template modeling show that, due to lack of constraints in a few cases, comparative modeling is not easy to perform. For these specific cases, a hybrid method of modeling using multiple CDR templates could be a good strategy. Similarly, this study also emphasizes the need for different tools to assess V_H_H models. Interestingly, the generation of the models still shows an excellent correlation between the value of the DOPE score and the quality of the model (compared to the experiential structure in terms of RMSD). Similarly, the analysis of the models selected using PROSA software [47] shows that the models have compatibility scores very close to those of the solved structures, even in the case of models that are difficult to build (see Table A1). In addition to RMSD for the analysis of the entire domain or its different regions, a protein block-based scoring function may be useful for analysis of local conformation.

In summary, this study had shown that, often, a multi-template is the best method to obtain, on average, a correct V_H_H model, and that DOPE score is a relevant measure to select this model. However, it also shows that, for some V_H_Hs, it is difficult to propose satisfactory models. In the same way, the use of the structurally closest V_H_H is not always the best choice, underlying the possibility for future improvement.

## 4. Materials and Methods

### 4.1. Dataset

V_H_H structures were retrieved from the Protein Databank (https://www.rcsb.org/, accessed on 31 September 2018) [12] using keywords ‘VHH’ and ‘Nanobody’. Only V_H_H structures without missing residues and/or modified ones were selected. After a structural inspection, a final redundancy filter (of 95% of sequence identity) was applied.

### 4.2. Sequence Alignment

In order to (i) compute the sequence identity and (ii) to prepare files for the comparative modeling (see details below), pairwise sequence alignments were performed using Clustal Omega (v 1.2.4) [48].

### 4.3. Structural Similarity of Protein Structures and Structural Models

The root mean square deviation (RMSD) was used locally to quantify the structural similarity between two aligned protein structures. ProFit (http://www.bioinf.org.uk/programs/profit/, accessed on 31 September 2018) based on the McLachlan algorithm for the superposition [49] was used in the study to calculate RMSD.

### 4.4. Comparative Modeling of V_H_H

Comparative modeling was performed using Modeller software [24,25], one of the most popular comparative modeling approaches, and also the most widely used for V_H_H molecular models [21]. Modeller 9 v.16 was used to model V_H_H query sequences. As input, an alignment file in the prescribed format of the query with the target template(s) was generated. The best structural model was chosen using the DOPE score implemented in Modeller to rank the models [26,50].

Four different strategies to address different hypotheses were conceived:

- In the first scenario, sequence identity between the query and template sequences is used as a criterion to select a template that shares the best sequence identity with each query sequence (namely scenario *bestSeqIdTemp*). It is the most classical protocol for template selection used in homology modeling,
- In the second scenario, templates are selected using structural similarity (to already solved structures of query sequences) measured using RMSD as criterion (namely scenario *bestStructTemp*). The template that had the lowest RMSD with the experimentally resolved structure of the query was chosen. This scenario is not possible in factual sense and it is a theoretical assessment, to check the maximal accuracy reachable with the closest structural template.
- The third scenario is based on a multi-template strategy. Three templates exhibiting the highest sequence identity with the query were selected (namely scenario *3bestSeqIdTemp*). In a multiple template mode, better models are expected thanks to the combination of different structures.
- The last scenario is also a theoretical case to access the maximal reachable accuracy using all possible templates (namely scenario *All*). Indeed, all previous scenarios had a specific a priori. Here, all structures were used independently as potential templates. It permits us to have more insights that would have been missed in previous scenarios such as; could another V_H_H template, other than the best sequence identity, and the best structurally close ones provide a better structural model.

### 4.5. Local Conformational Analysis

Secondary structure assignment was performed using DSSP 2015 version 2.2.1 [51] with default parameters [52]. Similarly we used also protein blocks (PBs [22]); they are a structural alphabet composed of 16 local prototypes [23]. Each PB is characterized by a series of 8 φ, ψ dihedral angles of five consecutive residues. Each PB assignment focuses on the central residue. PBs give a reasonable approximation of all local protein 3D structures [53]. They are labelled from *a* to *p*. PBs *m* and *d* can be roughly described as prototypes for α-helix and central β-strand, respectively. PBs *a* to *c* primarily represent β-strand N-caps and PBs *e* and *f* representing β-strand C-caps; PBs *g* to *j* are specific to coils; PBs *k* and *l* to α-helix N-caps while PBs *n* to *p* to α-helix C-caps. PB assignment was carried out using PBxplore tool (available at GitHub) [39].

The equivalent number of PBs (*N_eq_*) is a statistical measure similar to entropy. It represents the average number of PBs for a residue at a given position, which is calculated as follows [22]:

(1) $$N_{eq}=\exp(-\overset{16}{\underset{x=1}{\sum }}f_{x\;}ln\;f_{x})$$

where, *f_x_* is the probability of PB *x*. A *N_eq_* value of 1 indicates that only one type of PB is observed, while a value of 16 is equivalent to a random distribution.

To detect a change in PBs profile, a Δ*PB* value was calculated [44]. It corresponds to the absolute sum of the differences for each PB between the probabilities of a PB *x* to be present in the first and the second structures (*x* goes from PB *a* to PB *p*). Δ*PB* is calculated as follows:

(2) $$\Delta PB=\;\overset{16}{\underset{x=1}{\sum }}\left|\left(f_{x}^{1}-\;f_{x}^{2}\right)\right|$$

where, *f*^1^*_x_* and *f*^2^*_x_* are the percentages of occurrence of a PB *x* in the analyzed structures. A value of 0 indicates perfect PB identity, while a score of 2 indicates a total difference.

### 4.6. Protein Structure and Structural Model Visualization

Visualization of models and/or original structures were performed using PyMOL Version 1.7.2 [54,55].

### 4.7. Scripting

All scripts for analyzing V_H_H structures and models were done using Python 3.6 [56] with NumPy library [53] and R 3.3.3 [57].

## Figures and Tables

**Table 1:** Structural models with more than 3Å RMSD. Listed below are the PDB IDs and chain IDs of the V_H_H query (column 1) with corresponding RMSD values of DOPE score selected models for the three scenarios (columns 2 to 4), the sequence identity (or mean sequence identity for the multi-template case) with the structural template (columns 5 to 7, SI and MSI), and the RMSD with the structural templates for scenario 1 (column 8) and scenario 2 (column 9). The scenario in which the best model has the highest RMSD is marked in red for scenario 1, green for scenario 2, and blue for scenario 3. A ‘*’ indicates the lowest RMSD value.

Query	RMSD SC1 (Å)	RMSD SC2 (Å)	RMSD SC3 (Å)	SI SC1 (%)	SI SC2 (%)	MSI SC3 (%)	RMSD T SC1 (Å)	RMSD T SC2 (Å)
3G9A:B	7.87	3.46 *	3.89	69.17	59.63	68.2	4.63	1.82
3SN6:N	6.87	6.87	3.05 *	83.03	83.03	78.7	1.42	1.42
3EAK:B	5.59	1.39 *	1.64	67.86	66.39	67.2	1.89	1.44
5E7B:A	5.52	4.01 *	6.21	75.63	70.53	74.1	4.04	1.56
4C57:C	5.49	1.78 *	4.13	67.86	54.1	67.0	2.13	1.74
4IDL:A	5.13	2.72 *	3.18	67.26	60.17	65.9	3.01	1.27
5F1O:B	4.28	6.62	2.09 *	76.47	68.75	74.4	3.91	1.08
1KXV:D	4.08	2.00 *	4.47	64.96	63.33	64.1	2.89	2.00
5GXB:B	3.94	1.74 *	4.01	78.13	69.04	77.3	3.21	1.43
5BOP:C	3.91	1.36 *	1.97	78.63	70.24	78.0	2.46	1.14
4HEP:G	3.81	5.09	3.60 *	77.23	63.47	75.1	3.06	1.84
5F7L:B	3.29	1.34 *	2.66	76.72	71.42	75.5	1.90	1.01
4WEN:B	3.28	0.99	0.68 *	75.63	74.38	74.7	3.23	0.97
4WGV:D	3.04	2.49 *	2.94	75.83	64.34	74.9	2.86	1.73
4EIZ:D	2.93	6.89	2.37 *	76.78	73.21	76.3	3.14	1.12
3K3Q:A	2.86	5.96	2.55 *	56.19	50.00	55.1	2.03	1.71
2X1O:B	2.57	4.73	2.53 *	74.17	69.64	73.6	2.86	1.32
1I3U:A	2.06	4.31	2.29 *	65.85	61.46	64.7	1.66	1.53
4LAJ:H	2.29	4.30	2.04 *	79.67	65.21	78.6	2.11	1.60
2X6M:A	2.51	3.94	1.60 *	70.58	64.34	69.9	3.12	1.25
4GRW:F	2.43	3.77	1.97 *	79.67	71.42	76.8	2.11	1.54
3K81:B	2.74	2.45 *	3.41	72.50	65.81	71.5	2.80	1.91

## Data Availability

Data available on request.

## Supplementary Materials

The following are available online at https://www.mdpi.com/article/10.3390/ijms22189771/s1.

## Appendix A

**Table A1:** Prosa II Z -Score values for the best DOPE models selected. Column 1: sequence from the PDBID:chainID used. Column 2, 3, 4, and 5: Z-score value of the selected model from scenario 1, scenario 2, scenario 3, and solved crystal structure, respectively.

	Prosa II Z Scores for 22 Difficult Cases			
	SC1	SC2	SC3	Crystal Structure
1I3U:A	−4.98	−5.37	−5.36	−5.53
1KXV:D	−4.08	−6.4	−4.41	−4.73
2X1O:B	−6.06	−5.64	−5.68	−5.72
2X6M:A	−6.01	−5.98	−5.8	−6.84
3EAK:B	−5.45	−5.63	−5.81	−5.97
3G9A:B	−4.51	−4.75	−4.14	−5.79
3K3Q:A	−6.12	−5.87	−5.89	−5.22
3K81:B	−4.91	−5.11	−5.21	−5.44
3SN6:N	−4.02	−4.05	−5.21	−5.14
4C57:C	−5.54	−5.02	−5.00	−5.80
4EIZ:D	−6.94	−6.01	−6.05	−6.56
4GRW:F	−5.76	−5.78	−6.52	−6.6
4HEP:G	−5.48	−6.19	−5.98	−6.59
4IDL:A	−4.40	−4.25	−5.18	−4.30
4LAJ:H	−5.94	−5.84	−6.45	−6.72
4WEN:B	−4.93	−6.09	−6.15	−6.4
4WGV:D	−4.95	−4.78	−5.21	−5.04
5BOP:C	−5.97	−6.09	−6.12	−6.04
5E7B:A	−5.58	−6.12	−5.99	−6.16
5F1O:B	−5.77	−6.42	−6.59	−6.71
5F7L:B	−6.43	−5.78	−5.57	−5.66
5GXB:B	−6	−6.15	−5.75	−5.35
